# Seventh Councilor (Austin) District Medical Society

**Published:** 1908-01

**Authors:** 


SEVENTH COUNCILOR (AUSTIN) DISTRICT MEDICAL SOCIETY.—Annual meeting, Austin, December 19. Papers: "Opsonic Work"; "Wright's Therapeutics," by Dr. H. N. Graves, Georgetown (published herewith). "A Critical Review of Ehrlich's Chemical Theory," by Dr. J. W. McLaughlin, Sr. "Race Suicide," by Dr. Geo. H. Harwood, Johnson City. "Human and Bovine Tuberculosis," by Dr. W. J. Mathews. Dr. McLaughlin's excellent paper was criticised and opposed by Prof. Henry Winston Harper, Professor of Chemistry, University of Texas, and discussed by others, and the battle between the two critics was most thrilling. It was a "dog fall." The meeting was very interesting and was well attended. The officers for 1908 are Dr. Homer Hill, President (the Presidents of the component county medical societies are ex-officio vice-presidents of the District Society). Dr. W. A. Harper was elected Secretary.

The meeting closed with a swell banquet at the Café Royale, where wine, wit and eloquence flowed to the strains of "celestial" (gramophone) music.

Dr. F. E. Daniel was toastmaster, and the following are the toasts proposed by him and responded to as per program.

TOASTS.

"The University of Texas and Its Great Medical School: The Incubator of the Next Generation of Philosophers, Scientists and Statesmen."

"A little learning is a dangerous thing,
Drink deep or taste not the Pierian spring."

Response by Dr. J. W. McLaughlin, our representative on the Board of Regents and late Professor of Medicine in the University Medical College.

"The Texas State Medical Association. A Grand Body of Earnest Workers in the Cause of Humanity. May Its Meetings

Always Be Harmonious, and Its Councils Guided by Wisdom and Moderation."

"In union there is strength."

Response by Dr. W. J. Mathews, President Austin Board of Health.

"The Ideal Physician, the Highest Type of American Citizenship."

"A man he was, to all the country dear,
And passing rich with fifty pounds a year."

Response by Dr. J. C. Anderson, of Granger.

"The Combination Physician and Surgeon; the All-Around 'Specialist'; Peculiarly an American Product."

"And still they gazed, and still the wonder grew,
That one small head could carry all he knew."

Response by Dr. Z. T. Bundy, Surgeon to Soldiers' Home, where he practices everything but obstetrics.

"The Ambitious Doctor."

"The heights by great men reached and kept
Were not attained by sudden flight.
But they, while their companions slept,
Were toiling upward, in the night."

Response by Dr. H. B. Granberry, Retiring President Travis County Medical Society.

"The Doctor's Sweetheart.—She May Not Always Be His Wife; But the Doctor's Wife Should Always Be His Sweetheart."

"'Tis sweet to hear the faithful watchdog's honest bark
Bay deep-mouthed welcome as we draw near home.
'Tis sweet to know there is an eye will mark
Our coming, and grow brighter when we come."

Response by Councilor T. J. Bennett of the Bully Seventh.

"Woman—God's Best Gift to Man."

"Oh woman, in thine hour of ease,
Uncertain, coy and hard to please;
As fickle as the glimmering shade
By the trembling leaf of the Aspen made;
But when pain and anguish rack the brow,
A ministering angel thou!"

Response by "Horace" N. Graves, the Sweet Singer of Georgetown.

Visions of pompano and terrapin, venison and quail; and of champagne, and claret and Chartreuse will abide with the fellows, we hope, until next annual.

